# Radical-pair-based magnetoreception in birds: radio-frequency experiments and the role of cryptochrome

**DOI:** 10.1007/s00359-017-1189-1

**Published:** 2017-06-13

**Authors:** Christine Nießner, Michael Winklhofer

**Affiliations:** 1 Ernst Strüngmann Institute for Neuroscience, Deutschordenstr 46, 60528 Frankfurt am Main, Germany; 2 Institute for Biology and Environmental Sciences IBU, School of Mathematics and Science, University of Oldenburg, 26111 Oldenburg, Germany

**Keywords:** Radical-pair mechanism, Cryptochrome, Inclination compass, Radio-frequency magnetic fields, Larmor frequency

## Abstract

The radical-pair hypothesis of magnetoreception has gained a lot of momentum, since the flavoprotein cryptochrome was postulated as a structural candidate to host magnetically sensitive chemical reactions. Here, we first discuss behavioral tests using radio-frequency magnetic fields (0.1–10 MHz) to specifically disturb a radical-pair-based avian magnetic compass sense. While disorienting effects of broadband RF magnetic fields have been replicated independently in two competing labs, the effects of monochromatic RF magnetic fields administered at the electronic Larmor frequency (~1.3 MHz) are disparate. We give technical recommendations for future RF experiments. We then focus on two candidate magnetoreceptor proteins in birds, Cry1a and Cry1b, two splice variants of the same gene (Cry1). Immunohistochemical studies have identified Cry1a in the outer segments of the ultraviolet/violet-sensitive cone photoreceptors and Cry1b in the cytosol of retinal ganglion cells. The identification of the host neurons of these cryptochromes and their subcellular expression patterns presents an important advance, but much work lies ahead to gain some functional understanding. In particular, interaction partners of cryptochrome Cry1a and Cry1b remain to be identified. A candidate partner for Cry4 was previously suggested, but awaits independent replication.

## Introduction

Birds use their magnetic compass sense to extract directional information from the Earth’s magnetic field (Wiltschko and Wiltschko 1972). With a “magnetic map sense”, they can moreover locate their position (Kishkinev et al. 2015), which together with compass, information is required for magnetic navigation. Here, we focus on the basic principles of functionality of the magnetic compass in birds and on experiments that have been designed to test for the underlying biophysical mechanism.

The magnetic compass in birds is the so-called inclination compass (Wiltschko and Wiltschko 1972), which detects the spatial orientation of the field lines but not their polarity. The functional window of the compass sense is adapted to the naturally occurring geomagnetic field strengths, but can be adjusted to ten times lower field strengths (Winklhofer et al. 2013). The avian magnetic inclination compass is light-dependent, requiring wavelengths between UV and green light (Wiltschko and Wiltschko 2005), and it is localized in the eye (Semm et al. 1984; Semm and Demaine 1986; Wiltschko et al. 2002, 2003; Mouritsen et al. 2005; Liedvogel et al. 2007; Heyers et al. 2007; Stapput et al. 2010; Zapka et al. 2009). The characteristics of the magnetic compass in birds—polarity insensitivity and light-dependence—are consistent with a photo-activated radical pair (Schulten et al. 1978; Schulten 1982; Ritz et al. 2000; recent review: Hore and Mouritsen 2016) and has guided the search for a molecule that acts as a magnetic field detector when activated by light and that resides in the eye, leading to cryptochromes as structural candidate.

## Radical-pair mechanism

A free radical by definition is a chemical species with an unpaired valence electron. A well-known example from cell biology is the signaling agent nitric oxide ·(NO), which has 5 + 6 = 11 valence electrons, one of which is unpaired. A free radical is a paramagnetic entity, because the electron spin of the unpaired electron is unpaired (uncompensated). However, the magnetic moment of a free radical is many orders of magnitude too small to significantly interact with the comparatively weak Earth’s magnetic field. The situation is different for two radicals originating from the same process, either by bond splitting from the same parent (e.g., hydrogen peroxide decomposition into HO· and ·OH) or by a redox reaction, where an electron is transferred from a donor to an acceptor. In such a geminate radical pair, the electron spins are in a coherent spin state, oriented either antiparallel to each other (singlet state) or parallel to each other (triplet state), and their relative orientation, the so-called spin multiplicity, can be affected even by a moderate-strength external magnetic field (Schulten et al. 1976). A radical pair is relatively short-lived and may recombine by electron back transfer, but only if it is in the correct spin state (Schulten et al. 1976). Spin selectivity of geminate radical-pair recombination, therefore, is essential for the chemical magnetoreception pathway proposed by Schulten et al. (1978), who also pointed out that it provides a natural explanation for the magnetic compass sense of song birds being insensitive to the polarity of the magnetic field lines (Wiltschko and Wiltschko 1972).

Soon afterwards, Schulten (1982) suggested a photoexcitation pathway for the formation of the precursor state from which the geminate radical pair descends and thereby redefined the concept of physico-chemical light-dependent magnetoreception that had been proposed by Leask (1977) to occur through an optical pumping pathway. However, it was not until two decades later that a concrete candidate molecule—cryptochrome—was suggested (Ritz et al. 2000) and which then acted as a game changer for research into radical-pair-based magnetoreception. For details about the radical-pair mechanism and how it could be realized in the avian magnetoreception pathway, we refer to the excellent review by Hore and Mouritsen (2016). We here discuss physical concepts only to the point where they are essential for grasping the idea behind experiments conducted with radio-frequency magnetic fields, which are the key diagnostic test for radical-pair-based magnetoreception.

### Effects of broadband magnetic fields

Schulten et al. (1976) emphasized the importance of hyperfine interactions for singlet to triplet interconversion in a geminate radical pair. Hyperfine interaction refers to the magnetic interaction between electron spin and nuclear spin. In a spin-correlated radical pair where each unpaired electron experiences different hyperfine interactions, the two spins precess at different frequencies and thus change their relative orientation, oscillating between singlet and triplet states. This natural interconversion between singlet and triplet is modulated by an external magnetic field (Schulten et al. 1978). Moreover, the singlet–triplet interconversion can be perturbed by an oscillating magnetic field whose frequency matches any of the interconversion frequencies in the radical pair system. The hyperfine splitting frequencies are in the lower MHz radio-frequency (RF) range, but not exactly known yet for the biological radical pair underlying the magnetic sense (Ritz et al. 2004). In a behavioral experiment, this limitation can be overcome by applying an RF magnetic field over a frequency spectrum that covers the lower MHz range. Indeed, European robins subjected to broadband-noise RF magnetic fields were disoriented (Ritz et al. 2004), a key finding that was independently replicated with significantly weaker RF magnetic field intensities: Engels et al. (2014) and Schwarze et al. (2016) reported that RF magnetic field intensities of the order of 1 nT (root mean square) were already sufficient for disrupting orientation. At the theoretical level, it is not understood how such low RF levels could affect a radical pair (e.g., Kavokin 2009, 2017; Hore and Mouritsen 2016) or could have unspecific physiological effects on cells, tissues, or the whole organism. An unequivocal effect of RF magnetic fields on the partitioning of reactive oxygen species has been demonstrated experimentally for very high RF intensities of 20,000 nT (Usselman et al. 2016), but it is not clear if the radical pair mediating that effect has sufficiently long spin-correlation time for the effect to still occur at RF intensities of 20 nT or lower. Likewise, RF-induced heating of magnetic material in a magnetic-particle-based magnetoreception pathway is possible for high RF magnetic field levels (Shcherbakov and Winklhofer 2010), but exceedingly unlikely to occur under 1 nT RF magnetic fields.

### Effects of Larmor frequency RF magnetic fields

Thalau et al. (2005) and Ritz et al. (2009) conducted a series of experiments aimed at perturbing a radical-pair-based compass with a magnetic field oscillating at a special RF frequency, the so-called Larmor frequency of the free electron, defined as:

$$f_{L} = \gamma B ,$$

where *γ* = 28,024.95 MHz/T is the gyromagnetic ratio for a “free” electron (i.e., *g-*factor of 2.0023) and *B* is the absolute value of the external magnetic field in units of T (flux density). The rationale for applying this frequency is that it matches the Zeeman splitting induced by the static geomagnetic field (Thalau et al. 2005). Both studies were conducted in Frankfurt in a local magnetic field of between 46,000 and 47,400 nT intensity, with an RF frequency of 1.315000 MHz, i.e., the Larmor frequency for *B* = 46,922.5 nT. Birds turned out to be disoriented in the Larmor condition down to RF amplitudes of 15 nT, while 150 nT administered at non-Larmor frequencies had no effect. These results have been interpreted in terms of a radical pair in which one of the radicals is devoid of hyperfine interactions and thus is only sensitive to the external magnetic field (Ritz et al. 2009). However, the work of Ritz et al. (2009) has also triggered critical reviews (Kirschvink et al. 2010; Kavokin et al. 2014). Kirschvink et al. (2010) criticized the lack of confound control in RF experiments. An obvious confound would be the usage of two different test chambers, one dedicated to test, the other to controls, so that a statistical meaningful difference between test and control could be due as well to the differences between the test chambers. Equally confounding is electronic equipment that is running during test trials only (Kirschvink et al. 2010). It is clear that the comparison between test and control will be more meaningful if the overall design of the conditions is similar. Schwarze et al. (2016) implemented a confound control to ascertain that birds from both the experimental group (RF) and control group (sham-RF) experienced otherwise identical conditions in the test setup, housed in an electromagnetically shielded environment. Specifically, the RF amplifier was operating under load for the whole duration of both control and test trials. The vibrations and acoustic noise produced by the cooling fans of the amplifier then is of comparable magnitude and frequency content, no matter whether it delivers electric power into the coil (RF) or into a dummy load (sham-RF). Schwarze et al. (2016) did not observe complete disorientation in the birds tested under the Larmor condition, that is, the level of non-orientation (*p* value 0.138) was much lower than the one reported by Ritz et al. (2009) or by the other independent replication study by Kavokin et al. (2014). Importantly, the orientation data under Larmor-RF were statistically indistinguishable from those under sham-RF conditions (Schwarze et al. 2016). From a statistical point of view, the large number of birds (31 per group) tested by Schwarze et al. (2016) implies high statistical power and thus a low probability for false negatives, i.e., for not identifying a potentially existing difference between experimental and sham-RF group. Even at higher RF amplitude, in an RF condition similar to Thalau et al. (2005), Schwarze et al. (2016) obtained a significant orientation at the group level.

It is unclear why the Larmor-RF condition affected complete disorientation in Frankfurt (Thalau et al. 2005; Ritz et al. 2009) and at the Curish spit (Kavokin et al. 2014), but not in Oldenburg, where the most rigorous RF test design was implemented (Schwarze et al. 2016). In Table 1, we have juxtaposed the Larmor frequency experiments in terms of experimental parameters, RF equipment, group sizes, and statistical results. The studies differed in various experimental details (coil design, RF equipment, illumination), bird testing procedures, and analysis protocols. All these differences make it difficult to identify the key factor responsible for the presence or absence of disorientation effects under the local Larmor condition. It is not even clear if the local Larmor-RF condition was always exactly matched in each experimental trial so as to produce the maximum resonance effect. If a 25 nT single-frequency magnetic field is to have an effect on the spin dynamics in a radical pair, it has to act upon the spin-correlated pair for longer than 200 μs (Hiscock et al. 2016), which implies that its resonance frequency is sharper than 5 kHz, ca. 0.5% in terms of the absolute frequency (5/1315). The 0.5% figure defines the accuracy with which the absolute value of the magnetic field has to be measured at the test site before setting the Larmor frequency (see below).

**Table 1:** Comparison of experiments testing songbirds for disorientation in monofrequent radio-frequency (RF) magnetic fields at the local Larmor frequency of the free electron

Study	[1], [2]	[3]	[4]
Location	Frankfurt, Germany	Courish Spit, Russia	Oldenburg, Germany
Bird species	*Erithacus rubecula*	*Sylvia borin*	*Erithacus rubecula*
Local magnetic induction (nT)	Between 46,000 and 47,400	50,100	48,600 ± 240
Local Larmor frequency (MHz)	Between 1.289 and 1.328	1.404	1.362 ± 0.007
RF test condition	[1] 485 nT@1.315 MHz [2] 15 nT@1.315 MHz	190 nT@1.403 MHz	(a) 400 nT@1.363 MHz (b) 48 nT@1.363 MHz
Confound control (sham-RF)	None	None	(a) 400 nT@ 50 Hz (b) RF power fed into dummy load
Birds per condition	[1] 12 (spring), 16 (autumn) [2] 12	8	(a) 19, 20 (b) 31
Trails per bird in each condition	3	3	10
Rayleigh test (second order, RF cond)	[1] *p* > 0.05 (n.s.) [2] *p* > 0.05 (n.s.)	*p* = 0.83 (n.s.)	(a) *p* < 0.01 (**) (b) *p* = 0.138 (n.s.)
MWW: RF vs ctrl	[1] *p* < 0.001 (***) [2] *p* < 0.001 (***)	*p* = 0.025 (*)	(a) *p* = 0.41 (n.s) (b) *p* = 0.77 (n.s)
RF generator	Stanford Research DS340	Not specified	Rigol DG1022
Frequency standard	None	None	None
RF power amplifier	Amplifier Research 25 W 1–1000 MHz	Not specified	TOMCO, 50 W CW 0.1–20 MHz
Spectrum analysis	HP 89410A (DC—10 MHz)	Digital oscilloscope FFT	Rohde and Schwarz FSV-3 (10 Hz–3.6 GHz)
Background RFlevel	Not reported	*B*_rms_ < 0.5 nT	Figure 3 in [4], *E*_rf_ and *B*_rf_
Emlen funnel	Plastic (PVC)	Cardboard	Nonmagnetic metal (aluminum)
Trial duration	75 min	40 min	60 min
Light conditions	Monofrequent 565 nm 2.1 mW/m^2^	Blurred night sky (outdoor)	White (incandescent bulb) 2.1 mW/m^2^

Study: [1] Thalau et al. (2005); [2] Ritz et al. (2009); [3] Kavokin et al. (2014); [4] Schwarze et al. (2016)

Larmor frequency refers to the “free” electron with a g-factor of 2.0023

Rayleigh test: test against null hypothesis that directions were randomly drawn from a uniform distribution over the unit circle; low *p* value suggests that null hypothesis is false, i.e., that mean direction is significant

MWW (Mardia–Watson–Wheeler test): non-parametric test against null hypothesis that both experimental and control distribution are drawn from the same distribution, low *p* value suggests that null hypothesis is false, i.e., that the distributions are not the same

## Recommendations for future RF experiments

Below, we give some recommendations for improving RF studies at the Larmor frequency or at other specific frequencies that remain to be identified for avian cryptochrome in silico or in vitro.

### Magnetometer calibration

To determine the Larmor frequency in the local magnetic field, the total magnetic field intensity needs to be measured at the test site with an accuracy of better than 250 nT (0.5% of the total magnetic field). The expected local field intensity according to the geomagnetic reference field may, in some places, differ by more than 100 nT from the actual local field. Hence, there is no substitute for an on-site measurement. The ideal device for absolute measurement of magnetic field intensity is a proton precession magnetometer, because it is drift free. In contrast, flux gate magnetometers drift, but when calibrated before the experiment and kept at constant temperature, they serve the purpose as well. The proton magnetometer is also a good indicator of an unrecognized magnetic field gradient, in the presence of which it does not tune.

### Frequency calibration and standards

It is clear that Larmor frequency experiments also require accurate frequency control. On modern digital signal generators, the frequency can be set with great precision, but its accuracy depends on the accuracy of the internal frequency reference of the generator, which may have drifted away (aging effect) since the last instrumental calibration. The same holds true for a signal/spectrum analyzer or for a frequency counter. The previous studies obviously used the internal frequency reference of the signal generator, but made no mention of frequency calibration. That point is less critical for new instruments delivered with calibration certificate but may be an issue with old equipment. For future studies on the Larmor frequency, it would be ideal to provide signal generator (and analyzer) with an accurate external reference frequency from a rubidium frequency standard (i.e., 10 MHz Rubidium oscillator).

### Suppression of harmonics

When working with a monochromatic RF field to probe a specific Zeeman or hyperfine transition in the radical-pair compass, it is particularly important to have a clean RF signal. However, signal generators and amplifiers shift a considerable fraction of RF power into multiples of the set frequency (“harmonics”) when the amplification level is too high. Harmonics can be largely suppressed with electronic LC-filter circuits or when using RF coils tuned to a narrow frequency band (Malkemper et al. 2015).

### Magnetic field monitoring

Usually the RF field in the test chamber is measured only before or after the experiment, but not during the whole duration of the experiment. RF transients from erratic sources may influence test animals (Engels et al. 2014) and should, therefore, be monitored continuously using a passive loop antenna in the setup.

The static magnetic field near the test site should also be monitored over the course of the experiment to capture erratic local changes in the magnetic field. Global fluctuations of the geomagnetic field (due to geomagnetic storms) may also affect a Larmor experiment. A look at geomagnetic observatory data (intermagnet.org) could help to identify critical time periods.

### Funnel material

Aluminum funnels are not ideal for RF experiments, because they shield RF fields to some extent. According to our own measurements (MW) in the electromagnetically shielded test facility in Oldenburg (Schwarze et al. 2016), using a 25 mm diameter passive H-field probe (LF-R400, Langer EMV, Bannewitz, Germany), the spectral magnetic field intensity in the lower half of an aluminum funnel is attenuated by 9.3 dB (by a factor of 2.9) for the vertical component and by 5.9 dB (by a factor of 1.96) for the horizontal field of the RF magnetic field. Thus, an RF field of 48 nT measured without an aluminum funnel then translates into at least 16 nT in the aluminum funnel, which would still be enough to produce complete disorientation according to Ritz et al. (2009).

## Cryptochrome

The flavoprotein cryptochrome has been proposed as a candidate photomagnetoreceptor protein in the seminal paper by Ritz et al. (2000). It belongs to a class of proteins that are known to host radical pairs formed by light-induced electron transfer (Giovani et al. 2003; Biskup et al. 2009). Cryptochromes are related to the photolyases, that is, both have the Photolyase Homology Region (PHR) domain, which includes the binding sites for the chromophore flavin, the light sensitive co-factor of the protein. Partch et al. (2005) provided biochemical evidence that light activation of cryptochrome effects a conformational change that exposes the C-terminal domain with its putative signal transduction domain.

Cryptochromes were first discovered in plants by Ahmad and Cashmore (1993) and subsequently in many other organisms. A great deal has been learnt about the structure, functions, occurrence, and the degree of relationship of cryptochromes (see, e.g., Cashmore et al. 1999; Sancar 2003; van der Horst and Hellingwerf 2004; Oztürk et al. 2009; Liedvogel and Mouritsen 2010; Chaves et al. 2011; Müller and Ahmad 2011; Solov’yov et al. 2012). Cryptochromes are common in plants, bacteria, and many different animals including cubozoa (box jellyfish), insects, and vertebrates like fish, amphibians, mammals, and birds (see, e.g., Cashmore et al. 1999; Sancar 2003; Liedvogel and Mouritsen 2010; Haug et al. 2015). The functions markedly differ between the different organisms and between the cryptochromes. In plants, cryptochromes, for example, control the growth of the hypocotyl or transition to flowering (Ahmad and Cashmore 1993; Lin et al. 1998; for summary see Lin 2002). In vertebrates, some cryptochromes are involved in circadian rhythms (Cashmore 2003; Sancar 2003; Oztürk et al. 2007; Chaves et al. 2011). In the monarch butterfly, cryptochromes are involved in the adjustment of the sun compass (Zhu et al. 2008; Kyriacou 2009). In the plant Arabidopsis (Ahmad et al. 2007, but see Harris et al. 2009; for review, see Maffei 2014), and among animals in *Drosophila* (Yoshii et al. 2009; Fedele et al. 2014) and cockroaches (Bazalova et al. 2016), there is a link between magnetic sensitivity and cryptochromes.

Here, we concentrate on the cryptochromes as the potential receptor molecules for the magnetic compass in birds. Various behavioral experiments and neuronal investigations have pinpointed the magnetic compass of birds in the eye (Semm et al. 1984; Semm and Demaine 1986, but see Ramirez et al. 2014; Mouritsen et al. 2005; Liedvogel et al. 2007; Heyers et al. 2007; Zapka et al. 2009; Stapput et al. 2010). Below, we focus on what we know about the precise localization and characteristics of cryptochromes located in the retina of birds. Birds commonly have four different cryptochromes: two splice variants of Cry1 (Cry1a and Cry1b), Cry2, and Cry4 (Bailey et al. 2002; Haque et al. 2002; Mouritsen et al. 2004; Möller et al. 2004; Watari et al. 2012; Mitsui et al. 2015). Cry2 includes a sequence that suggests a nuclear location (Möller et al. 2004; Mouritsen et al. 2004), from where it is unlikely to generate a nerve signal. Hence, Cry2 is not the first choice in the study of potential receptor molecule for the magnetic compass (Liedvogel and Mouritsen 2010).

In 2004, Möller and colleagues described the structure of Cry1b of European robins, and at the same time, Mouritsen and colleagues published the sequence of Cry1 in garden warblers (Möller et al. 2004; Mouritsen et al. 2004). Möller et al. (2004) discussed Cry1b as a potential receptor molecule for the magnetic compass because of its bird-specific C-Terminus, in contrast to the C-Terminus of Cry1a that mammals also have. Mouritsen et al. (2004), in addition to sequencing the Cry1, analyzed the location of garden warbler retinal Cry1 and studied nocturnal vs diurnal expression levels, using commercial Cry1 antiserum (which we now know does not discriminate between Cry1a and Cry1b). In the garden warbler retina, Cry1 is expressed in the inner segments of the photoreceptors, in the ganglion cells, and in the displaced ganglion cells of the inner nuclear layer, in which immediate–early gene expression indicated high levels of neuronal activity at night (Mouritsen et al. 2004). Cry1 expression in the night-migrating garden warbler is high both at day and night, whereas Cry1 expression in the corresponding retinal cells of the non-migratory zebra finch is high at daytime and drops to unspecific background levels at night (Mouritsen et al. 2004). It was concluded that Cry1 could be the receptor molecule of the magnetic compass, because garden warblers need to have the receptor molecule also at night (see also Liedvogel and Mouritsen 2010). Also Fusani et al. (2014) reported that Cry1 expression depends on the level of migratory restlessness (*Zugunruhe*) and not on species-specific or seasonal factors. It should be noted that day active birds also use the magnetic compass for orientation (see Wiltschko and Wiltschko 2007).

In 2016, three independent studies could show that Cry1b is localized in the retinal ganglion cells of different bird species (Bolte et al. 2016; Nießner et al. 2016a; Ströckens and Güntürkün 2016), but the three studies discussed the function of this bird-specific splice variant of Cry1b differently. Bolte et al. (2016) observed Cry1b antibody staining exclusively in the cytosol of retinal ganglion cells, displaced ganglion cells, and inner segments of photoreceptors, but not in the nucleus where circadian-activity cryptochromes would be expected, and concluded that the cellular context of Cry1b would allow for the possible involvement in a radical-pair-based magnetoreception mechanism. On the other hand, Nießner et al. (2016a) found Cry1b in the retinal ganglion cells of European robins only during the migration phase; outside this phase, Cry1b expression was reduced to unspecific background levels. They concluded that Cry1b could be involved in circa-annual rhythms and is rather not suitable as receptor molecule for sensing magnetic directions, because the avian magnetic compass shows no seasonal variation (see also Wiltschko and Wiltschko 2007). Ströckens and Güntürkün (2016) found Cry1b in pigeons already in the embryonic state before the rods develop and suggested that Cry1b could be involved in the induction of asymmetries in the visual system.

Cry1a was localized along membrane stacks of the outer segments of the ultraviolet/violet-sensitive (UV/V-) cones of European robins and chickens (Nießner et al. 2011). In Western blots, Cry1a occurred in both the membrane and cytosolic fraction (Nießner et al. 2011). Since cryptochrome itself is a water-soluble protein without membrane domain, the presence of Cry1a in the membrane fraction implies a membrane-bound partner. Indeed, if Cry1a is to act as a compass molecule, its rotational motion needs to be restrained to some extent by anchoring it to a larger cellular structure such as the cell membrane (Schulten 1982; Lau et al. 2012). However, the molecular identity of the binding partner remains to be elucidated.

In some mammals, Cry1 (the mammalian homologue protein to bird Cry1a) was also detected specifically in the blue-sensitive cones that are homologues to the UV/V-cones of birds (Nießner et al. 2016b). In the Carnivora families Canidae, Mustelidae, and Ursidae and in some Primates, Cry1 is localized along the disk membranes of the outer segments of these cones; the blue-sensitive cones of the other studied mammals do not show such Cry1 expression. Mammals are also able to use the Earth’s magnetic field for orientation, but at the moment, we do not know enough about the mechanism(s) of this compass system to speculate about an involvement of Cry1 (see Begall et al. 2014; Malkemper et al. 2015; Nießner et al. 2016b). One specific feature of the immunolabeling technique used to detect Cry1a in birds and also Cry1 in mammals should be mentioned. The antibody used in the studies by Nießner and colleagues binds to Cry1a only when the retina has been exposed to short wavelength light before. The Cry1a antibody, therefore, appears to detect the protein in a conformation that is induced by charge reorganization after light absorption in its FAD chromophore. This has also been observed for Cry4 (Watari et al. 2012).

Behavioral studies show that the magnetic compass in birds works with the light from UV to green (Wiltschko and Wiltschko 2001), and Cry1a is activated under all these light conditions (Nießner et al. 2013). In experiments where the birds were deprived of such light before the tests, they cannot orient magnetically and the immunolabeling shows no light-activated Cry1a in the cones (Nießner et al. 2014; Wiltschko et al. 2014). These results are consistent with the characteristics of the magnetic compass.

Qin et al. (2016) have focused on Cryptochrome 4 (Cry4), which in the house sparrow brain has non-rhythmic expression (Helfer et al. 2006), and identified the iron-sulfur protein IscA1 (termed MagR) as a possible interaction partner of Cry4 in a helical superstructure. However, the study has been criticized on various grounds (Winklhofer and Mouritsen 2016; Hochstoeger et al. 2016). Another issue is the immunohistochemical detection of Cry4 in the retina of pigeons with an antiserum that was raised against the full-length pigeon Cry4 protein. Given the considerable sequence homology among the cryptochromes, the antiserum possibly could have stained all cryptochromes in the retina. Nevertheless, Cry4 remains an interesting magnetoreceptor candidate. As opposed to Cry1a and Cry1b, the precise localization of Cry4 in the avian retina is not known yet.

At present, there is no compelling evidence for the involvement of any Cry in bird magnetoreception. RNA interference experiments will be useful to identify which Cry is necessary in the light-dependent magnetoreception pathway. Still, the ultimate challenge will be to demonstrate whether or not Cry acts as primary magnetoreceptor. The action of Cry may be restricted to signal transduction in the dark via protein interaction cascades, as surmised recently by Kutta et al. (2017), who have shown that vertebrate cryptochromes have a low binding affinity for the flavin co-factor. It is too early, though, to discount a magnetoreceptive function sensu strictu for vertebrate cryptochromes without knowing their interaction partners, because the argument by Kutta et al. (2017) applies to the protein in isolation. Therefore, it will be essential to elucidate interaction partners of Cry1a, 1b, and Cry4 before their functions can be constrained further.

## References

[CR1] Ahmad M, Cashmore AR (1993). HY4 gene of *A. thaliana* encodes a protein with characteristics of a blue-light photoreceptor. Nature.

[CR2] Ahmad M, Galland P, Ritz T, Wiltschko R, Wiltschko W (2007). Magnetic intensity affects cryptochrome-dependent responses in *Arabidopsis thaliana*. Planta.

[CR3] Bailey MJ, Chong NW, Xiong J, Cassone VM (2002). Chickens’ Cry2: molecular analysis of an avian cryptochrome in retinal and pineal photoreceptors. FEBS Lett.

[CR4] Bazalova O, Kvicalova M, Valkova T, Slaby P, Bartos P, Netusil R, Tomanova K, Braeunig P, Lee HJ, Sauman I, Damulewicz M, Provaznik J, Pokorny R, Dolezel D, Vacha M (2016). Cryptochrome 2 mediates directional magnetoreception in cockroaches. Proc Natl Acad Sci USA.

[CR5] Begall S, Burda H, Malkemper EP (2014). Magnetoreception in mammals. Adv Stud Behav.

[CR6] Biskup T, Schleicher E, Okafuji A, Link G, Hitomi K, Getzoff ED, Weber S (2009). Direct observation of a photo-induced radical pair in a cryptochrome blue-light photoreceptor. Angew Chem Int.

[CR7] Bolte P, Bleibaum F, Einwich A, Günther A, Liedvogel M, Heyers D, Depping A, Wöhlbrand L, Rabus R, Janssen-Bienhold U, Mouritsen H (2016). Localisation of the putative magnetoreceptive protein cryptochrome 1b in the retinae of migratory birds and homing pigeons. PLoS One.

[CR8] Cashmore AR (2003). Cryptochromes: enabling plants and animals to determine circadian time. Cell.

[CR9] Cashmore AR, Jarillo JA, Wu YJ, Liu D (1999). Cryptochromes: blue light receptors for plants and animals. Science.

[CR10] Chaves I, Pokorny R, Byrdin M, Hoang N, Ritz T, Brettel K, Essen LO, van der Horst GT, Batschauer A, Ahmad M (2011). The cryptochromes: blue light photoreceptors in plants and animals. Annu Rev Plant Biol.

[CR11] Engels S, Schneider NL, Lefeldt N, Hein CM, Zapka M, Michalik M, Elbers D, Kittel A, Hore PJ, Mouritsen H (2014). Anthropogenic electromagnetic noise disrupts magnetic compass orientation in a migratory bird. Nature.

[CR12] Fedele G, Green EW, Rosato E, Kyriaco CP (2014). An electromagnetic field disrupts negative geotaxis in Drosophila via a CRY-dependent pathway. Nat Comm.

[CR13] Fusani L, Bertolucci C, Frigato E, Foà A (2014). Cryptochrome expression in the eye of migratory birds depends on their migratory status. J Exp Biol.

[CR14] Giovani B, Byrdin M, Ahmad M, Brettel K (2003). Light-induced electron transfer in a cryptochrome blue-light photoreceptor. Nat Struct Biol.

[CR15] Haque R, Chaurasia SS, Wessel JH, Iovome PM (2002). Dual regulation of cryptochrome 1 mRNA expression in chicken retina by light and circadian oscillators. NeuroReport.

[CR16] Harris SR, Henbest KB, Maeda K, Pannell JR, Timmel CR (2009). Effect of magnetic fields on cryptochrome-dependent responses in *Arabidopsis thaliana*. J R Soc Interface.

[CR17] Haug MF, Gesemann M, Lazovic V, Neuhauss SCF (2015). Eumetazoan cryptochrome phylogeny and evolution. Genome Biol Evol.

[CR18] Helfer G, Fidler AE, Vallone D, Foulkes NS, Brandstaetter R (2006). Molecular analysis of clock gene expression in the avian brain. Chronobiol Int.

[CR19] Heyers D, Manns M, Luksch H, Güntürkün O, Mouritsen H (2007). A visual pathway links brain structures active during magnetic compass orientation in migratory birds. PLoS One.

[CR20] Hiscock HG, Kattnig DR, Maholopoulus DR, Hore PJ (2016). Floquet theory of radical pairs in radiofrequency magnetic fields. J Chem Phys.

[CR21] Hochstoeger T, Nimpf S, Keays DA (2016). ISCA1 and CRY4: An improbable proposition. BioRxiv.

[CR22] Hore PJ, Mouritsen H (2016). The radical-pair mechanism of magnetoreception. Annu Rev Biophys.

[CR23] Kavokin K (2009). The puzzle of magnetic resonance effect on the magnetic compass of migratory birds. Bioelectromagnetics.

[CR24] Kavokin K (2017). Can a hybrid chemical-ferromagnetic model of the avian compass explain its outstanding sensitivity to magnetic noise?. PLoS One.

[CR25] Kavokin K, Chernetsov N, Pakhomov A, Bojarinova J, Kobylkov D, Namozov B (2014). Magnetic orientation of garden warblers (Sylvia borin) under 1.4 MHz radiofrequency magnetic field. J R Soc Interface.

[CR26] Kirschvink JL, Winklhofer M, Walker MM (2010). Biophysics of magnetic orientation: strengthening the interface between theory and experimental design. J R Soc Interface.

[CR27] Kishkinev D, Chernetsov N, Pakhomov A, Heyers D, Mouritsen H (2015). Eurasian reed warblers compensate for virtual magnetic displacement. Curr Biol.

[CR28] Kutta RJ, Archipowa N, Johannissen LO, Jones LO, Scrutton NS (2017). Vertebrate cryptochromes are vestigial flavoproteins. Sci Rep.

[CR29] Kyriacou C (2009). Clocks, cryptochromes and Monarch migrations. J Biol.

[CR30] Lau JC, Rodgers CT, Hore PJ (2012). Compass magnetoreception in birds arising from photo-induced radical pairs in rotationally disordered cryptochromes. J Roy Soc Interface.

[CR31] Leask MJM (1977). A physicochemical mechanism for magnetic field detection by migratory birds and homing pigeons. Nature.

[CR32] Liedvogel M, Mouritsen H (2010). Cryptochromes—a potential magnetoreceptor: what do we know and what do we want to know?. J R Soc Interface.

[CR33] Liedvogel M, Feenders G, Wada K, Troje NF, Jarvis ED, Mouritsen H (2007). Lateralized activation of cluster N in the brains of migratory songbirds. Eur J Neurosci.

[CR34] Lin C (2002). Blue light receptors and signal transduction. Plant Cell.

[CR82] Lin C, Yang H, Guo H, Mockler T, Chen J, Cashmore AR (1998). Enhancement of blue-light sensitivity ofArabidopsis seedlings by a blue light receptor cryptochrome 2. Proc Natl Acad Sci USA.

[CR35] Maffei ME (2014). Magnetic field effects on plant growth, development, and evolution. Front Plant Sci.

[CR36] Malkemper EP, Eder SHK, Begall S, Phillips JB, Winklhofer M, Hart V, Burda H (2015). Magnetoreception in the wood mouse (*Apodemus sylvaticus*) influence of weak frequency-modulated radio frequency fields. Sci Rep.

[CR37] Mitsui H, Maeda T, Yamaguchi C, Tsuji Y, Watari R, Kubo Y, Okano K, Okano T (2015). Overexpression in yeast, photocycle, and in vitro structural change of an Avian putative magnetoreceptor cryptochrome4. Biochemistry.

[CR38] Möller A, Sagasser S, Wiltschko W, Schierwater B (2004). Retinal cryptochrome in a migratory passerine bird: a possible transducer for the avian magnetic compass. Naturwissenschaften.

[CR39] Mouritsen H, Janssen-Bienhold U, Liedvogel M, Feenders G, Stalleicken J, Dirks P, Weiler R (2004). Cryptochrome and activity markers co-localize in bird retina during magnetic orientation. Proc Natl Acad Sci USA.

[CR40] Mouritsen H, Feenders G, Liedvogel M, Wada K, Jarvis ED (2005). Night-vision brain area in migratory songbirds. Proc Natl Acad Sc USA.

[CR41] Müller P, Ahmad M (2011). Light-activated cryptochrome reacts with molecular oxygen to form a flavin-superoxide radical pair consistent with magnetoreception. J Biol Chem.

[CR42] Nießner C, Denzau S, Gross JC, Peichl L, Bischof HJ, Fleissner G, Wiltschko W, Wiltschko R (2011). Avian ultraviolet/violet cones identified as probable magnetoreceptors. PLoS ONE.

[CR43] Nießner C, Denzau S, Stapput K, Ahmad M, Peichl L, Wiltschko W, Wiltschko R (2013). Magnetoreception: activated cryptochrome 1a concurs with magnetic orientation in birds. J R Soc Interface.

[CR44] Nießner C, Denzau S, Peichl L, Wiltschko W, Wiltschko R (2014). Magnetoreception in birds: I. Immunohistochemical studies concerning the cryptochrome cycle. J Exp Biol.

[CR45] Nießner C, Denzau S, Malkemper EP, Gross JC, Burda H, Winklhofer M, Peichl L (2016). Cryptochrome 1 in retinal cone photoreceptors suggests a novel functional role in mammals. Sci Rep.

[CR46] Nießner C, Gross JC, Denzau S, Peichl L, Fleissner G, Wiltschko W, Wiltschko R (2016). Seasonally changing cryptochrome 1b expression in the retinal ganglion cells of a migrating passerine bird. PLoS One.

[CR47] Oztürk N, Song SH, Ozgür S, Selby CP, Morrison L, Partch C, Zhong D, Sancar A (2007). Structure and function of animal cryptochromes. Cold Spring Harb Symp Quant Biol.

[CR48] Oztürk N, Selby CP, Song SH, Ye R, Tan C, Kao YT, Sancar A (2009). Comparative photochemistry of animal type 1 and type 4 cryptochromes. Biochemistry.

[CR49] Partch C, Clarkson MW, Özgür S, Lee AL, Sancar A (2005). Role of structural plasticity in signal transduction by the cryptochrome blue-light photorezeptor. Biochemistry.

[CR50] Qin S, Yin H, Yang C, Dou Y, Liu Z, Zhang P, Yu H, Huang Y, Feng J, Hao J, Hao J, Deng L, Yan X, Dong X, Zhao Z, Jiang T, Wang HW, Luo SJ, Xie C (2016). A magnetic protein biocompass. Nat Mater.

[CR51] Ramirez E, Marin G, Mpodozis J, Letlier JC (2014). Extracellular recordings reveal absence of magneto sensitive units in the avian optic tectum. J Comp Physiol A.

[CR52] Ritz T, Adem S, Schulten K (2000). A model for photoreceptor-based magnetoreception in birds. Biophys J.

[CR53] Ritz T, Thalau P, Phillips JB, Wiltschko R, Wiltschko W (2004). Resonance effects indicate a radical-pair mechanism for avian magnetic compass. Nature.

[CR54] Ritz T, Wiltschko R, Hore PJ, Rodgers CT, Stapput K, Thalau P, Timmel CR, Wiltschko W (2009). Magnetic compass of birds is based on a molecule with optimal directional sensitivity. Biophys J.

[CR55] Sancar A (2003). Structure and function of DNA photolyase and cryptochrome blue-light photoreceptors. Chem Rev.

[CR56] Schulten K, Treusch J (1982). Magnetic field effects in chemistry and biology. Festkörperprobleme.

[CR57] Schulten K, Staerk H, Weller A, Werner HJ, Nickel B (1976). Magnetic field dependence of the geminate recombination of radical ion pairs in polar solvents. Z Physik Chem.

[CR58] Schulten K, Swenberg CW, Weller A (1978). 1. A biomagnetic sensory mechanism based on magnetic field modulated coherent electron spin motion. Z Physik Chem.

[CR59] Schwarze S, Schneider NL, Reichl T, Dreyer D, Lefeldt N, Engels S, Baker N, Hore PJ, Mouritsen H (2016). Weak broadband electromagnetic fields are more disruptive to magnetic compass orientation in a night-migratory songbird (*Erithacus rubecula*) than strong narrow-band fields. Front Behav Neurosci.

[CR60] Semm P, Demaine C (1986). Neurophysiological properties of magnetic cells in the pigeon’s visual system. J Comp Physiol A.

[CR61] Semm P, Nohr D, Demaine C, Wiltschko W (1984). Neural basis of the magnetic compass: interaction of visual, magnetic and vestibular inputs in the pigeon’s brain. J Comp Physiol A.

[CR62] Shcherbakov VP, Winklhofer M (2010). Theoretical analysis of flux amplification by soft magnetic material in a putative biological magnetic-field receptor. Phys Rev E.

[CR63] Solov’yov IA, Domratcheva T, Shahi ARM, Schulten K (2012). Decrypting cryptochrome: revealing the molecular identity of the photoactivation reaction. J Am Chem Soc.

[CR64] Stapput K, Thalau P, Wiltschko R, Wiltschko W (2010). Magnetoreception of directional information in birds requires nondegraded vision. Curr Biol.

[CR65] Ströckens F, Güntürkün O (2016). Cryptochrome 1b: a possible inducer of visual lateralization in pigeons?. Eur J Neurosci.

[CR66] Thalau P, Ritz T, Stapput K, Wiltscho R, Wiltschko W (2005). Magnetic compass orientation of migratory birds in the presence of a 1.315 MHz oscillating field. Naturwissenschaften.

[CR67] Usselman RJ, Chavarriaga C, Castello PR, Procopio M, Ritz T, Dratz EA, Singel DJ, Martino CF (2016). The quantum biology of reactive oxygen species partitioning impacts cellular bioenergetics. Sci Rep.

[CR68] Van der Horst MA, Hellingwerf KJ (2004). Photoreceptor proteins, “star actors of modern times”: a review of the functional dynamics in the structure of representative members of six different photoreceptor families. Acc Chem Res.

[CR69] Watari R, Yamaguchi C, Zemba W, Kubo Y, Okano K, Okano T (2012). Light-dependent structural change of chicken retinal cryptochrome 4. J Biol Chem.

[CR70] Wiltschko W, Wiltschko R (1972). Magnetic compass of European robins. Science.

[CR71] Wiltschko W, Wiltschko R (2001). Light-dependent magnetoreception in birds: the behaviour of European robins, Erithacus rubecula, under monochromatic light of various wavelengths and intensities. J Exp Biol.

[CR72] Wiltschko W, Wiltschko R (2005). Magnetic orientation and magnetoreception in birds and other animals. J Comp Physiol A.

[CR73] Wiltschko W, Wiltschko R (2007). Magnetoreception in birds: two receptors for two different tasks. J Ornithol.

[CR74] Wiltschko W, Traudt J, Güntürkün O, Prior H, Wiltschko R (2002). Lateralization of magnetic compass orientation in a migratory bird. Nature.

[CR75] Wiltschko W, Munro U, Hugh F, Wiltschko R (2003). Lateralisation of magnetic compass orientation in Silvereyes, *Zosterops lateralis*. Australian J Zool.

[CR76] Wiltschko R, Gehring D, Denzau S, Nießner C, Wiltschko W (2014). Magnetoreception in birds: II. Behavioural experiments concerning the cryptochrome cycle. J Exp Biol.

[CR77] Winklhofer M, Mouritsen H (2016). A magnetic protein biocompass?. bioRxiv.

[CR78] Winklhofer M, Dylda E, Thalau P, Wiltschko W, Wiltschko R (2013). Avian magnetic compass can be tuned to anomalously low magnetic intensities. Proc R Soc B.

[CR79] Yoshii T, Ahmad M, Helfrich-Föster C (2009). Cryptochrome mediates light-dependent magnetosensitivity of Drosophila’s circadian clock. PLoS Biol.

[CR81] Zapka M, Heyers D, Hein CM, Engels S, Schneider NL, Hans J, Weiler S, Dreyer D, Kishkinev D, WildJM Mouritsen H (2009). Visual but not trigeminal mediation of magnetic compass information in a migratorybird. Nature.

[CR80] Zhu H, Sauman I, Yuan Q, Casselman A, Emery-Le M, Emery P, Reppert SM (2008). Cryptochromes define a novel circadian clock mechanism in monarch butterflies that may underlie sun compass navigation. PLoS Biol.

